# Immuno-Endocrinology of COVID-19: The Key Role of Sex Hormones

**DOI:** 10.3389/fendo.2021.726696

**Published:** 2021-12-02

**Authors:** Flavia Tramontana, Sofia Battisti, Nicola Napoli, Rocky Strollo

**Affiliations:** ^1^ Department of Medicine, Unit of Endocrinology & Diabetes, Università Campus Bio-Medico di Roma, Rome, Italy; ^2^ Radiology Department, Azienda Unità Sanitaria Locale (AUSL) Romagna M. Bufalini Hospital, Cesena, Italy; ^3^ Istituto Scientifico Romagnolo per lo Studio e la Cura dei Tumori (IRST) Istituto di Ricovero e Cura a Carattere Scientifico (IRCCS), Meldola, Italy; ^4^ Dipartimento di Medicina Specialistica, Diagnostica e Sperimentale (DIMES), Alma Mater Studiorum-Universita di Bologna, Bologna, Italy; ^5^ Department of Science and Technology for Humans and the Environment, Università Campus Bio-Medico di Roma, Rome, Italy

**Keywords:** COVID-19, sex hormones, immuno-endocrinology, immune response, estrogen

## Abstract

Epidemiological evidence shows clear gender disparities in the Coronavirus 2019 Disease (COVID-19) severity and fatality. This may reflect the contribution of gender-related factors, such as sex hormones, to COVID-19 pathogenesis. However, the mechanism linking gender disparities to COVID-19 severity is still poorly understood. In this review, we will pinpoint several elements involved in COVID-19 pathogenesis that are regulated by the two main sex hormones, estrogen and androgen. These include tissue specific gene regulation of SARS-CoV2 entry factors, innate and adaptive immune responses to infection, immunometabolism, and susceptibility to tissue injury by cytopathic effect or hyper-inflammatory response. We will discuss the mechanistic link between sex hormone regulation of COVID-19 pathogenetic factors and disease severity. Finally, we will summarize current evidence from clinical studies and trials targeting sex hormones and their signalling in COVID-19. A better understanding of the role of sex hormones in COVID-19 may identify targets for therapeutic intervention and allow optimization of treatment outcomes towards gender-based personalised medicine.

## Introduction

The coronavirus disease 2019 (COVID-19) caused by the novel coronavirus SARS-CoV-2 represents a global health threat which has caused globally almost five million deaths by October 2021 (https://covid19.who.int/). Epidemiological evidence shows clear gender disparities in COVID-19 severity and fatality, placing gender as a main factor associated with a more severe disease, along with older age and cardiometabolic comorbidities. Although there is no significant sex difference in the proportion of individuals infected with SARS-CoV-2, males face double the risk of developing critical or fatal disease compared with females (1, 2). The sex gap is closed in prepubescent individuals, where both sexes are relatively protected from COVID-19 complications compared to adults (3, 4). This may reflect a possible contribution of gender-related factors, such as sex hormones, to COVID-19 pathogenesis (5, 6). However, out of 45 COVID-19 randomized controlled trials published by December 2020, only eight reported sex-disaggregated results or subgroup analyses (7).

In this review, we will pinpoint several elements involved in COVID-19 pathogenesis that are, at least partially, regulated by the two main sex steroids, estrogen and androgen. These include tissue specific gene regulation of SARS-CoV-2 entry factors, innate and adaptive immune responses to infection, immunometabolism, and susceptibility to tissue injury by cytopathic effect or hyper-inflammatory response. We will discuss the mechanistic link between sex-hormone regulation of COVID-19 pathogenetic factors and disease severity. Finally, we will summarize current evidence from clinical studies and trials targeting sex steroids and their signalling in COVID-19.

## Sex Hormones Control Virus-Host Interaction

SARS-CoV-2 is a single-stranded RNA-enveloped virus which uses the angiotensin-converting enzyme 2 (ACE2) as main access door to host cells. Entrance is facilitated by a host type 2 transmembrane serine protease, TMPRSS2, that is responsible for priming of the viral S glycoprotein. Increased tissue (co-)expression of ACE2 and TMPRSS2 at the virus entry sites may enhance infection, while downregulation may prevent SARS-CoV-2 binding to target cells. Both elements are under genetic control of sex steroids. ACE2 belongs to a subgroup of genes escaping X-chromosome inactivation with higher expression in men in several tissues (8), including a slight tendency for male-biased expression in the lung. The predominant male-biased expression of ACE2 is in line with the demonstrated higher ACE2 activity in males partially driven by sex steroids (9). Similarly, plasma ACE2 concentration has been found to be higher in men than in women possibly reflecting the expression at the tissue level (9). Sex steroids acts on the modulation of ACE2 expression in a tissue specific manner. According to studies in mice, estrogen receptor (ER) *alpha* activation by estradiol downregulates kidney ACE2 whereas ovariectomy, which is a state of estrogen deprivation, increased ACE2 activity and its expression in kidney and adipose tissue (10). Estrogen may also downregulate ACE2 in differentiated airway epithelial cells (11), the main SARS-CoV-2 entry site. It has been shown that ACE2 expression in primary isolated human airway smooth muscle (ASM) cells was lower in women compared to men, and significantly upregulated by testosterone (12). Furthermore, consistent with the age-dependent decline in circulating sex steroids, males experience lower ACE2 level than females in the late stage life (13).

Taken together, data suggest that the two main sex steroids produce opposite effects on ACE2 regulation. While estrogen tend to favour downregulation of the SARS-CoV-2 main receptor in several tissues, testosterone may enhance its expression.

TMPRSS2 is an androgen responsive gene (14). Its expression in human lung epithelial cells is upregulated by androgen while downregulated by androgen deprivation (15). Exogenous treatment with androgen was shown to be associated with an increased expression of TMPRSS2 in human type 2 pneumocytes (15). Therefore, part of the sex-based disparities in COVID-19 severity may be explained by high androgen in males that contribute to disease severity by promoting viral replication (16). In a study on 118 patients with primary prostate cancer, where *TMPRSS2* gene is a therapeutic target, androgen deprivation therapy was associated with a lower risk of SARS-CoV-2 infection (odds ratio 4.05; 95% CI 1.55–10.59). While these data needs validation in larger cohorts, they provide support to the association between androgen control of TMPRSS2 expression and risk of COVID-19. More recently, Samuel et al. performed a high-throughput screen with a library of 1443 FDA-approved drugs and a subsequent *in silico* screen of more than 9 million drug-like compounds to detect drugs effective in reducing ACE2 protein levels in cardiac cells and lung organoids. The authors found that the most effective drugs were linked to androgen receptor signalling inhibition (17). Inhibitors of 5-alpha reductase, which dampen androgen signalling, were able to downregulate both ACE2 and TMPRSS2 in lung epithelial cells and cardiac cells, leading to a lower SARS-CoV-2 infectivity in lung organoids (17).

Therefore, the increased (co-)expression of ACE2 and TMPRSS2 in SARS-CoV-2 target tissues may explain the higher occurrence of COVID-19 complications in males. However, whether sex hormones-dependent modulation of ACE2 or TMPRSS2 in the lung or other SARS-CoV-2 target tissues correlates with COVID-19 susceptibility or severity needs to be further elucidated.

## Sex Hormones Control Anti-Viral Immune Response

Gender is a key host factor influencing immune response, leading to differences in severity, prevalence, and pathogenesis of infection, with males generally more susceptible than females (18).

According to experimental evidence from the severe acute respiratory syndrome (SARS) caused by the SARS-CoV, another *beta*-coronavirus closely related to SARS-CoV-2, estrogen status is key in determining disease severity through modulation of the immune response. Ovariectomy of SARS-CoV infected female mice or treatment with an ER antagonist increased mortality compared to treatment with tamoxifen (a selective ER modulator) (19). The protective role of ER signalling has been linked to the induction of the “anti-viral status” mediated by type I interferon (IFN-I), a first line cytokine involved in host defence (4). In the SARS-CoV model, reduced survival was due to a robust viral replication and delayed IFN-I signaling which promoted accumulation of pathogenic inflammatory monocyte-macrophages, resulting in elevated lung pro-inflammatory cytokines and dysfunctional virus-specific T-cell responses (20). Thus, ER signalling may prime the IFN-I response and prevent viral replication. In contrast, a delayed IFN-I activation would generate a response-lag unable to compensate robust viral replication, thus leading to uncontrolled hyperinflammation. Data in humans provide support to this hypothesis. It has been shown that long-term treatment of post-menopause women with estradiol enhanced IFN-I response *via* the *toll-like* receptor (TLR) 7 pathway (21). TLR7 represents a sentinel receptor of the innate immune response to viral RNA from SARS-CoV-2 and other coronaviruses. Recognition of viral RNA by TLR7 expressed by dendritic cells triggers signalling cascades that result in the production of large amount of IFN-I. TLR7 is also sex biased as it is encoded by X chromosome and escapes X inactivation in B cells through epigenetic modifications (22). Thus, the increased expression of TLR7 in females compared to male can potentiate priming of IFN response by ER signalling, providing prompt antiviral defence and subsequent antibody production. Accordingly, it has been recently showed that loss of function in TLR7 gene resulted in a severe disease in young male patients after being infected with SARS-CoV-2 (23).

Estrogen can also modulate adaptive responses displaying a diphasic effect ranging from immunosuppressive at high concentration to immunostimulatory at lower concentration (19). For example, lymphocyte activation (e.g., proliferation and IFN-γ production) classically follows a diphasic dose-response to estrogen concentrations—low dose stimulation and high dose inhibition. Thus, in older women, the residual low levels of estrogen may up-regulate T cell IFN-γ, inducing effector T helper 1 proliferation and antibody production, all factors that sustain anti-viral immune responses. This may partially counterbalance the age dependent decline in adaptive immune responses (24).

Androgens present different effects on both innate and adaptive responses, which are often opposite to estrogens, and may explain the overall increased susceptibility to viral infections in males compared to females. First, testosterone is immune suppressive on dendritic cells (a main source of IFN-I) and reduces cytokine production by such cells, which is consistent with the reduced IFN-I response to TLR7 stimulation in males compared to females. Second, androgens inhibit T-helper 1 differentiation, thus potentially delaying the mounting of specific antiviral responses. Finally, testosterone directly enhances production of the immunosuppressive cytokine IL-10 by CD4^+^ T-cells, leading again to suppressed IFN-I response as well as impaired survival and differentiation of B cells (14). In a recent report of 136 SARS-CoV-2 PCR-positive patients, low testosterone and high estradiol were associated with disease severity in COVID-19 patients. Furthermore, both male and female COVID-19 patients presented elevated estradiol levels which positively correlated with plasma IFN-γ levels (25). However, it should be noted that men with acute or subacute illness are known to develop a transient functional secondary hypogonadism. Therefore, testosterone assessment at hospital admission may not reflect the real androgen status.

Therefore, the androgen and estrogen status can significantly affect immune response to viral infection. While estrogen promote the “anti-viral state” induced by IFN-I (innate immunity) and the development of anti-SARS-CoV-2 specific responses (adaptive immunity), androgen may delay the mounting of prompt and effective anti-viral response.

## Sex Hormones and Immunometabolism in COVID-19

Excess adiposity may provide and sustain a proinflammatory *milieu* that promotes an imbalanced immune response towards hyperinflammation. This may trigger a cytokine storm, leading to impaired T-cell anti-viral specific activity and exacerbate disease severity. Sex steroids have a clear role in shaping fat distribution. Males accumulate more visceral fat than females and age-related decline in sex steroids in humans is linked to greater fat accumulation in central regions. For example, downregulation of estrogen signalling through ER-alpha knockout lead to obesity in both male and female mice (26). Serum estrogen decline after menopause is associated with abdominal fat accumulation, while hormone replacement therapy reduces visceral fat (27), implying a key role of estrogen in regulating fat mass. Similarly, hypogonadism in men is associated with visceral adiposity while increasing testosterone concentration in men induces a reduction in total fat mass (28). However, while estrogens show beneficial effects on body fat regulation also in males, androgens have opposite effects in females. Women with polycystic ovary syndrome exhibit hyperandrogenism concomitant with visceral fat accumulation (29). Moreover, treatment with anabolic steroid having androgenic activity was associated with increased visceral fat accumulation (30). This is consistent with experimental evidence showing that treatment of female mice with testosterone results in greater body weight and fat mass that are sustained throughout adult life (31).

Female type fat distribution is associated with lower systemic inflammation, lower risk of developing cardiometabolic diseases and less severe COVID-19. We have recently shown that abdominal fat distribution characterized by increased visceral (VAT) and lower subcutaneous adipose tissue (SAT) is strongly associated with COVID-19 severity. SAT was higher in females than males, and inversely associated with the need of intensive treatment. Furthermore, each millimetre increase in VAT thickness increased risk of admission to intensive care unit by 16%, independently of body mass index (32). VAT has important immunological functions strongly contributing to the production of proinflammatory molecules such as IL-1β, IL-6, and TNF-α. One-third of the circulating IL-6 is produced by adipocytes and adipose tissue matrix (33). Low adiponectin and leptin resistance states associated with obesity display immune characteristics that partially resemble those seen in COVID-19 (34). In subjects with obesity, T-cell subpopulations (CD3+, CD4+, CD45RO+, CD8+) and their proliferative response to polyclonal mitogens are suppressed (35). These abnormalities are reversed with energy restriction (which decreases leptin) (36). In subjects with obesity, increased leptin levels correlate with circulating TNF-α, which displays a suppressive effect on lymphocytes count (35). This is in line with evidence suggesting that COVID-19 patients have a four-fold increase in leptin levels compared to non-infected controls (37). Adipokine levels are also under the control of sex steroids. Estradiol levels are directly associated with serum leptin while male steroids decrease leptin gene expression and secretion from human adipocytes (38). Conversely, low adiponectin levels in men *vs*. women appear to be predominantly mediated by male sex steroid hormones (39).

On the other hand, SARS-CoV-2 infection might enhance VAT inflammation. Mesenteric VAT, which surrounds the small intestine, is the first line of defence against pathogens translocated from the intestine to the circulation (40). Over 50% of COVID-19 patients test positive for SARS-CoV-2 RNA in stool, and 10% have gastrointestinal symptoms consistent with a SARS-CoV-2 cytopathic effect on enterocytes (41). According to single-cell RNA-sequencing data, the enterocyte is one of the main cells co-expressing high levels of the SARS-CoV-2 entry factors ACE2 and TMPRSS2 (42), suggesting that the gut may act as potential entry site of SARS-CoV-2. Virus recognition by the gut immune system may trigger an immunoinflammatory response spreading to mesenteric VAT and exacerbating local inflammation.

Therefore, the interaction between sex steroids, immune response and immuno-metabolic factors may generate an immunoendocrine environment that sustains infection and promotes COVID-19 progression at multiple levels.

## Sex Hormones and Sex Influence Vaccine Responses

COVID-19 vaccination campaign has started with a total of 6.5 billion vaccine doses that have been administered (https://covid19.who.int/info) and nearly 48% of the world population has received at least one dose of a COVID-19 vaccine (43). First reports on COVID-19 vaccine unfortunately were not powered to provide evidence of safety and efficacy by sex (44) although the point estimates of efficacy for subgroups was also high, consistent with that observed in the overall study population (45). However, evidence on other vaccines have shown differences in response or efficacy according to gender. For instance, the antibody response to seasonal influenza vaccines has been shown to be at least twice as high in females compared to males (18). A more robust protective antibody response that can facilitate vaccine efficacy in women was also observed after vaccination against influenza, hepatitis A and B, rubella, measles, mumps, herpes simplex and dengue viruses (46). This greater response may also explain why women experienced more frequent and severe adverse effects (18) as reported in the first month of the COVID-19 vaccine rollout (https://www.cdc.gov/mmwr). According to EUDRAVigilance report the suspected adverse drugs reaction of COVID-19 vaccines ranged from 59.0% to 72.0% in women and from 26.1% to 39.1% in men (https://www.adrreports.eu/en/). Mechanisms of these discrepancies may be related to differences in both innate and adaptive immunity as women have usually greater T cells activation, proliferation and cytotoxic activity as well as higher immunoglobulin basal levels and B cells number compared to men (47, 48). Moreover, studies in mice have demonstrated that while estrogen promote the development of antibodies testosterone may suppress it (47, 48). Indeed, a lower antibody response was observed to influenza vaccination in men compare to women, particularly in those with higher levels of testosterone at the time of vaccination (49). Despite this, only few studies have so far considered sex as a possible element that may affect COVID-19 vaccine response. Latest reports indicated a higher and similar efficacy in the vaccine arm compared to placebo for both men and women (50, 51). However, a recent meta-analysis including sex-disaggregated data from BNT162b2-BioNTech/Pfizer, mRNA-1273-Moderna, Ad26.COV2.S-Johnson&Johnson/Janssen showed a significantly increased efficacy in men compared to women. Males resulted to have a 33% reduced risk of developing COVID-19 compared to females (52). Data from a report on 248 healthcare workers undergoing the BNT162b2 vaccine showed a tendency for greater antibody response in females compared to males seven days after the second dose, although this difference was not significant (p=0.055) (53).

Current results are still controversial indicating that the efficacy of COVID-19 vaccines has not been adequately addressed in terms of sex and that the influence of sex and sex hormones is still poorly understood. Larger longitudinal studies are needed to clarify whether sex and sex steroids significantly affect the development of effective SARS-CoV-2 vaccine response.

## Sex Hormones Influence Inflammation and Susceptibility to Tissue Injury

Although there is no direct evidence available from studies carried out in SARS-CoV-2 infected subjects, literature data support the concept that sex steroids may influence susceptibility or protection to tissue injury of organs targeted by COVID-19 complications. The major morbidity and fatality from COVID-19 is due to acute viral pneumonitis that evolves to acute respiratory distress syndrome (ARDS) (54). This is characterised by hyaline membrane changes, microvessel thrombosis with exudative and proliferative phases of diffuse alveolar damage (55), sometimes superimposed by bacterial pneumonia. In a LPS-induced model of acute lung injury, male mice developed increased airway hyperresponsiveness and inflammation compared with their female counterparts (56). Treatment with testosterone enhanced inflammatory responses in females to a level that was similar to that showed in males. In contrast, gonadectomy reduced airway inflammation in males but not females suggesting that androgens sustain the proinflammatory action of LPS-induced lung insult (56). Ovariectomized females showed an increment in the neutrophil content in bronchoalveolar lavage fluids, myeloperoxidase activity in whole lung, and leak of albumin into the lung compared with intact females (57). However, estrogen replacement was found to be effective in reducing all these lung injury features by suppressing cell adhesion molecules and proinflammatory cytokines. In the carrageenan-induced pleurisy model, which represents a well-known murine model of inflammation, tissue damage was exacerbated by ER blockage (58). Several mechanisms may help to explain the protective effect of estrogen against acute lung injury and resolution of inflammation, including regulation of apoptosis (59) and nitric oxide production.

COVID-19 can also lead to a number of extrapulmonary manifestations (60). Among those, cardiovascular complications (myocardial dysfunction and arrythmias, acute coronary syndromes, and thrombotic complications) occur in over a third of hospitalised COVID-19 patients and are associated with a significant mortality risk (61). Direct cytopathic myocardial injury, systemic inflammation, virus-mediated endothelial damage, and hypoxia are some of the potential factors involved in these complications. Estrogen offers a vascular protective effect that may partially explain the gender discrepancy in COVID-19 deaths (62). Acute administration of estrogen in male rabbit have been shown to be protective against ischaemia, reducing infarct size by 20% (63, 64). Direct membrane signalling mediated by estrogen lead to vasodilation through nitric oxide release. Similarly, ER-alpha signalling mediated preservation of endothelial cell structure and function by preventing apoptotic pathway activation (65). Estrogen cardioprotective properties suggest that estrogen status may reduce susceptibility to cardiac injury, endotheliitis and subsequent cardiovascular complications associated with COVID-19 (60). However, direct evidence from COVID-19 studies is needed.

## What Is the Clinical Evidence for Estrogen and Anti-Androgenic Therapies in COVID-19?

Evidence that pharmacological modulation of estrogen and/or androgen signalling can prevent SARS-CoV-2 infection or disease severity is limited to a few observational studies. A retrospective study involving over 68,000 cases has studied the effect of exogenous estradiol administration on COVID-19 deaths. The authors found that death risk in women over 50 years of age receiving estradiol treatment was significantly reduced compared to those who were untreated (hazard ratio 0.29, 95% CI 0.11 to 0.76) (66). Montopoli et al. (14) observed that men treated with androgen depravation therapy for prostate cancer were protected against SARS-CoV-2 infection. Prostate cancer patients receiving androgen deprivation therapy had a significantly lower risk of SARS-CoV-2 infection compared with patients who did not receive it (4/5273 *vs*. 114/37161; odds ratio 4.05; 95% CI 1.55-10.59). In another observational study on 100 patients with androgenic alopecia and laboratory confirmed SARS-CoV-2 infection, treatment with dulasteride, which prevent testosterone conversion to dihydrotestosterone by inhibiting the 5-*alpha* reductase, was associated with a reduction in the frequency of clinical symptoms (67). In a double-blinded, randomized, prospective, investigational phase III study clinical trial involving 262 non hospitalized COVID-19 male patients (NCT04446429), the non-steroidal antiandrogen proxalutamide resulted in a reduction rate of hospitalization. Although such evidence provide support to the hypothesis that estrogen and androgen status are key players in COVID-19 pathogenesis and potential therapeutic targets, clinical evidence is limited by the small sample size and/or the observational nature of the findings. Seventeen clinical trials are registered on clinicaltrials.gov using as investigational product estrogen receptors agonists/modulators or anti-androgenic treatments in COVID-19 patients. Clinical trials are needed to define the role of such treatments for preventing COVID-19 severity and complications.

## Conclusions

**Figure 1:**
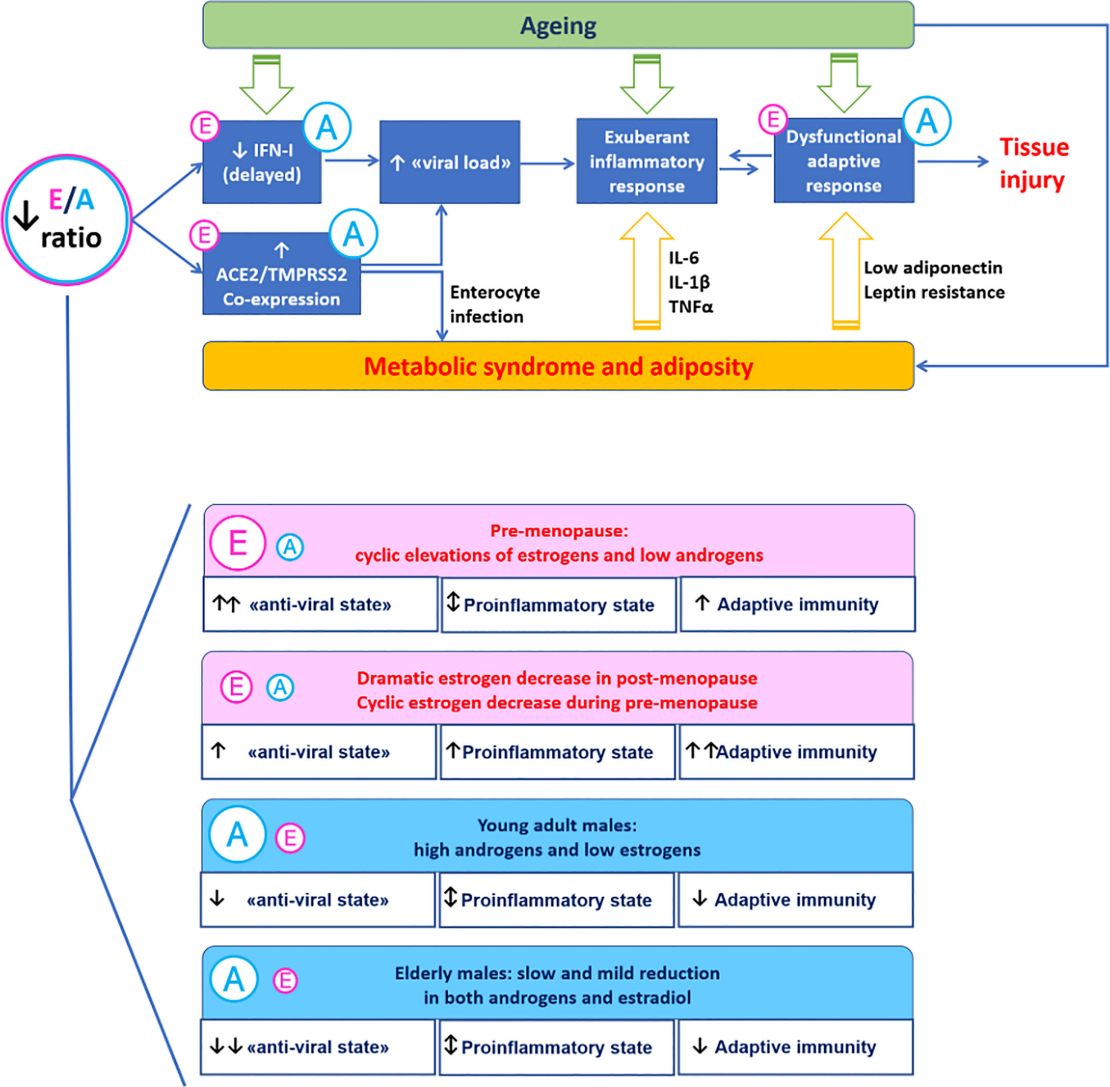
A mechanistic model for the immunoendocrinology of COVID-19. Differences in estrogen to androgen balance due to ageing and gender may modulate SARS-CoV-2 entry factors ACE2 and TMPRSS2 involved in virus-host interaction. Estrogen promote the “anti-viral state” induced by interferon type I (innate immunity) and the development of anti-SARS-CoV-2 specific responses (adaptive immunity). The proinflammatory *milieu* associated with excess visceral adiposity promotes SARS-CoV-2 infection and may be directly involved in the infection trough the enterocyte-adipose tissue axis.

The interaction of endocrine factors linked to gender provides a mechanism to explain at least in part the greater severity of COVID-19 in males compared to females. Androgen to estrogen balance may modulate virus-host interaction and immune response as estrogen enhance anti-viral defences and immune activity while androgen displays immunosuppressive action (**Figure 1**).

This leads not only to a greater immunity to virus infection observed in women compared to men but also may highlights a different response to vaccines between genders. Therefore, sex hormones status and other gender-related factors (biological and behavioural) may further modulate the risk of severe disease conferred by other risk factors such as ageing and cardiometabolic diseases. Whether sex steroids can provide a therapeutic option for COVID-19 is still unknown. Taken together, these data suggest that gender should be taken into account to optimize treatment outcomes for women and men towards gender-based personalized medicine.

## Author Contributions

RS and FT conceived the review and wrote the first draft of the manuscript. NN and SF participated in the conception of the review and revised it critically for important intellectual content. All authors contributed to the article and approved the submitted version.

## Conflict of Interest

The authors declare that the research was conducted in the absence of any commercial or financial relationships that could be construed as a potential conflict of interest.

## Publisher’s Note

All claims expressed in this article are solely those of the authors and do not necessarily represent those of their affiliated organizations, or those of the publisher, the editors and the reviewers. Any product that may be evaluated in this article, or claim that may be made by its manufacturer, is not guaranteed or endorsed by the publisher.
